# Role of Multimodality Imaging in Cardiac Sarcoidosis: A Retrospective Single-Center Experience

**DOI:** 10.3390/jcm13237335

**Published:** 2024-12-02

**Authors:** Vincent A. Torelli, Sanjay Sivalokanathan, Alexander Silverman, Syed Zaidi, Usman Saeedullah, Komail Jafri, James Choi, Luka Katic, Serdar Farhan, Ashish Correa

**Affiliations:** 1 Department of Cardiology, Icahn School of Medicine, Mount Sinai Morningside, New York, NY 10025, USA; 2 Lenox Hill Hospital, New York, NY 10075, USA

**Keywords:** cardiac sarcoidosis, CMR, FDG-PET, advanced cardiovascular imaging

## Abstract

**Background/Objectives:** Cardiac sarcoidosis (CS) is a rare entity characterized by granulomatous infiltration of the myocardium, which can lead to myocardial fibrosis, conduction abnormalities, and the development of heart failure, thereby elevating the risk of sudden cardiac death (SCD). While endomyocardial biopsy (EMBx) is regarded as the gold standard for diagnosis, its low sensitivity and inherent procedural risks may limit its practical application. **Methods:** This study retrospectively explored the role of advanced imaging modalities, specifically cardiovascular magnetic resonance imaging (CMR) and fluorodeoxyglucose positron emission tomography (FDG-PET), in the diagnosis and management of CS within a single center. In this retrospective study, we aimed to assess the utility of advanced imaging modalities in the clinical diagnosis of CS and the monitoring of treatment. **Results**: A total of 92 patients were identified as having cardiac sarcoidosis, with males constituting 66.3% of the sample and a mean age of 62 years (±11.9). Among these patients, 80 (87%) underwent FDG-PET. Here, the basal inferolateral segment was the most frequently observed segment of the heart with FDG uptake. A total of 77 patients (84%) underwent CMR, with 51 demonstrating late gadolinium enhancement (LGE). The basal inferolateral segment exhibited the highest frequency of LGE (26%). Logistic regression analysis indicated that patients presenting with a combination of LGE, FDG uptake on PET, and a “mismatch pattern” faced a two-fold increase in the risk of experiencing major adverse cardiac events (odds ratio = 2.311, *p* = 0.077). **Conclusions**: This study underscores the importance of multimodality imaging as a non-invasive alternative for CS diagnosis and management, reducing reliance on EMBx.

## 1. Introduction

Sarcoidosis is a complex and multifaceted disorder characterized by the formation of non-necrotizing epithelioid-cell granulomas, primarily located in the lungs, although it may manifest in multiple organs [1,2]. The prevalence of this condition exhibits considerable variability, with cardiac sarcoidosis (CS) representing a diagnostically challenging form. It is estimated that in 20% of sarcoidosis cases, there is cardiac involvement, with isolated cardiac involvement occurring in only 2–5% of individuals [3,4]. The granulomatous inflammation associated with CS disrupts the structural integrity of the myocardium, resulting in fibrosis and scarring. Such alterations can adversely affect ventricular function, lead to conduction disorders, and elevate the risk of sudden cardiac death (SCD) [2,5,6]. While endomyocardial biopsy (EMBx) can provide a definitive tissue diagnosis, it greatly lacks sensitivity due to the patchy involvement of the myocardium. The sensitivity of EMBx is <25%, though there may be some improvement with electroanatomic mapping, which is not easily available [2,5,6,7]. Given the diagnostic complexities inherent in CS, there is a compelling need to develop investigative strategies using non-invasive advanced cardiovascular imaging modalities. Developing such diagnostic strategies may facilitate early diagnosis, enhance risk stratification, and potentially transform the treatment paradigm.

Advanced imaging modalities, including cardiac magnetic resonance imaging (CMR) and fluorodeoxyglucose positron emission tomography (FDG-PET) computed tomography (CT), present non-invasive alternatives to EMBx for the assessment of myocardial involvement [8,9,10]. More importantly, these imaging techniques play a pivotal role in determining the extent of the disease and informing subsequent treatment decisions. While PET is important in identifying inflammation, CMR is useful in assessing myocardial fibrosis, edema, and perfusion defects. We describe our single-center experience of CS patients who underwent CMR and/or FDG-PET.

## 2. Materials and Methods

### 2.1. Study Design, Setting, Patient Enrollment, and Data Collection

In this observational cohort study, we retrospectively identified 93 patients within the Mount Sinai Morningside Electronic Medical Record System who exhibited diagnostic codes indicative of cardiac sarcoidosis. Duplicate records were eliminated, and we analyzed pertinent biological, radiological, and clinical data to ascertain cardiac sarcoidosis diagnoses in accordance with the Heart Rhythm Society (HRS) Diagnostic Criteria and the Japanese Circulation Society (JCS) [10]. In addition to the diagnostic codes, the inclusion criteria consisted of patients who exhibited biopsy-proven evidence of non-caseating granulomas from cardiac or extra-cardiac sources, alongside supporting features verified through cardiac imaging, as specified in the HRS guidelines. Furthermore, we included subjects whose conditions did not meet the HRS criteria for definitive or probable cardiac sarcoidosis (specifically, those who did not have positive cardiac/extra-cardiac biopsies) but possessed a relevant diagnostic code alongside both imaging findings, through either CMR or PET, and clinical features highly suggestive of cardiac sarcoidosis [11]. We categorized this group as “possible sarcoidosis”. A longitudinal database was established, encompassing data on specific laboratory and imaging findings, as well as clinical outcomes, which were systematically recorded. Only retrospective data were included, with data ranging from the patient’s initial presentation until October 2023.

### 2.2. Outcomes

The primary objectives of this study encompassed the results obtained from FDG-PET and CMR in patients diagnosed with CS. The focus was on identifying FDG patterns and the presence of late gadolinium enhancement (LGE). Secondary objectives included the collection of patient characteristics and examination of management trends, specifically highlighting the initiation rates of immunosuppression therapy and implantable cardioverter-defibrillator (ICD) implantation. Major adverse cardiac events (MACE) were defined as a documented decrease in left ventricular ejection fraction, as evaluated by transthoracic echocardiogram (TTE), the onset of right ventricular dysfunction, and the emergence of arrhythmias (such as right bundle branch block, first-degree atrioventricular block, complete heart block, ventricular tachycardia, ventricular fibrillation), or mortality.

### 2.3. Imaging Protocols

**Figure 1:**
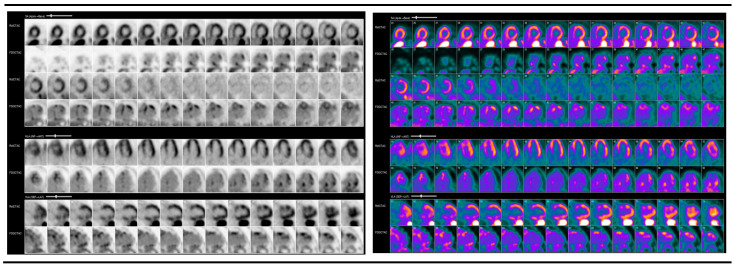
PET/CT demonstrating a “mismatch” pattern.

The protocol for PET/CT imaging necessitates a low-carbohydrate, high-fat diet prior to imaging studies to induce ketosis, thereby facilitating adequate physiological suppression of glucose uptake in the myocardium. While adherence to the dietary protocol was strongly encouraged, data on compliance were not recorded. Nonetheless, only participants who demonstrated adequate suppression, as indicated by their pre-imaging beta-hydroxy butyrate levels, were included in this study. Resting perfusion scans were obtained, after which patients received an injection of FDG, and subsequent scans were conducted 60 to 90 min post-injection. Segment-specific FDG uptake was documented using the American Heart Association (AHA) standardized 17-segment model, with the intensity of uptake quantified through standardized uptake values (SUV) [12,13]. The scans were evaluated for perfusion defects on resting scans, areas of FDG uptake after injection with FDG, and, in particular, “mismatch patterns” of FDG uptake corresponding to identified areas of perfusion deficit [12,14], which can be seen in Figure 1. CMR was performed in accordance with standard protocols, including late gadolinium enhancement (LGE), to assess for edema and fibrosis.

### 2.4. Statistical Analysis

Categorical variables are presented as total counts and percentages of the sample, while continuous variables are expressed as means accompanied by standard deviations. Statistical analysis was conducted utilizing JASP (ver 0.19.1.0) and Anaconda Python (ver 2024.10). Chi-squared analysis was employed to identify associations between variables, and logistic regression was used to assess differences, controlling for demographic and confounding factors such as age, sex, clinical characteristics, and pertinent medical history, including the presence of standard modifiable risk factors and concomitant ischemic cardiomyopathy. A significance level of *p* < 0.05 was established. Furthermore, a multinomial analysis with standardized residuals was performed to evaluate the proportional distribution of imaging findings. Standardized residuals with absolute values greater than 2 were deemed indicative of significant deviations from expected values. To assess concordance between PET/CT and CMR findings at each segment, a Cohen’s Kappa analysis was conducted. A Kappa value of 1 represents perfect agreement, whereas a value of −1 indicates no agreement beyond chance.

### 2.5. Missing Data

This study did not specifically impute or account for missing data. Variables with missing values were excluded from analysis on a per-variable basis, resulting in a complete-case analysis approach. For the primary outcomes and imaging data, missingness was minimal and did not exceed 5% for any critical variable, ensuring sufficient power and validity of the results. Given the retrospective nature, there were no subjects who were lost to follow-up.

## 3. Results

### 3.1. Patient Characteristics

A total of 93 patients were identified as having cardiac sarcoidosis, with 66.3% of the cohort being male. The mean age of the participants was 62 ± 11.9 years. Race was categorized as Caucasian, African American, Hispanic/Latino, or Other. Body mass index (BMI) was recorded for 72 individuals, yielding a mean value of 29 kg/m ± 5.6. Standardized modifiable risk factors for cardiovascular disease, including hypertension, hyperlipidemia, diabetes, and smoking, as well as prior medical histories such as myocardial infarction, percutaneous coronary intervention, coronary artery bypass grafting, and congestive heart failure, are detailed in Table 1.

### 3.2. FDG-PET Imaging Findings

**Figure 2:**
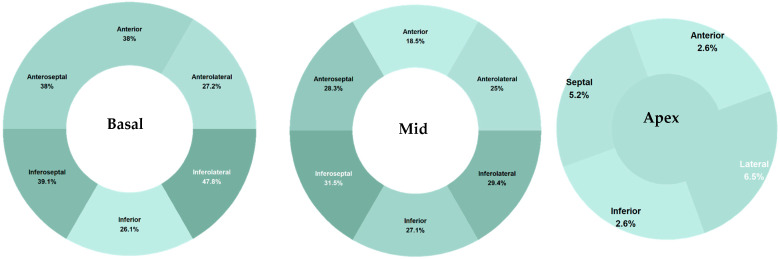
Heatmap detailing the FDG uptake pattern organized by the 17-segment model.

80 subjects underwent PET scan, as detailed in Table 2. Frequency analysis of FDG uptake by specific wall segments is depicted in Table 3, indicating that the basal inferolateral segment was observed most frequently, accounting for 47.83%, while the anterior apical segment was observed least frequently, at 7.61%. This is visually represented in Figure 2. Furthermore, statistical analysis revealed that the basal anterior, basal anteroseptal, basal inferoseptal, and basal inferolateral segments were significantly overrepresented, whereas the apical, apical lateral, apical inferior, apical anterior, and apical septal segments were found to be significantly underrepresented, as determined by multinomial analysis.

### 3.3. CMR Findings

**Figure 3:**
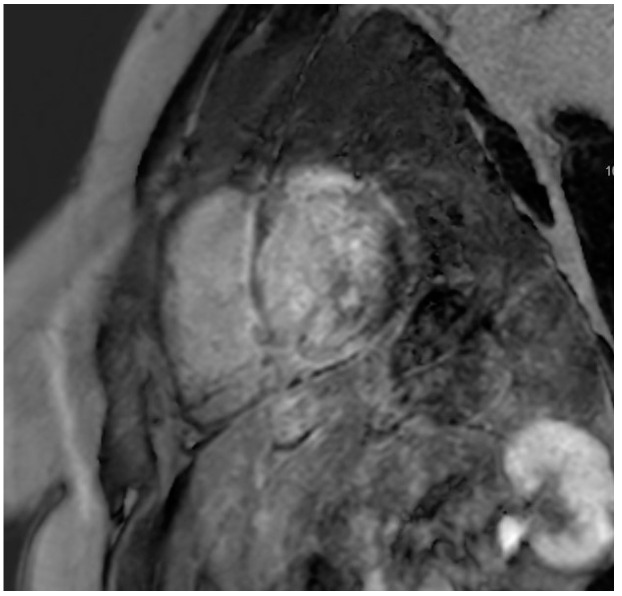
CMR demonstrating LGE localized to the basal regions.

**Figure 4:**
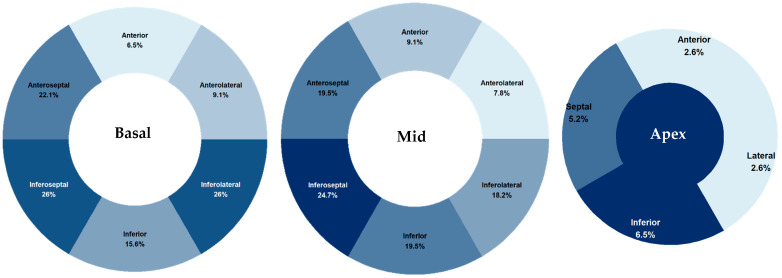
Heatmap detailing LGE distribution as assessed by CMR.

A total of 77 patients (84%) underwent CMR, with 51 patients exhibiting late gadolinium enhancement (LGE). The mean values for ejection fraction, end-systolic and end-diastolic volumes, stroke volume, and cardiac output are detailed in Table 4. A multinomial analysis of LGE uptake across specific wall segments is provided in Table 5. LGE in the basal inferolateral segment was observed with the highest frequency (26%), while the apical lateral and apical anterior segments were noted to be the least frequently affected, which can be visualized in Figure 3. Statistically significant overrepresentation was identified in the basal anteroseptal, basal inferoseptal, and basal inferolateral segments, in addition to the mid-anteroseptal, mid-inferoseptal, mid-inferior, and mid-inferolateral segments. Conversely, the mid-anterolateral segment and all apical segments were statistically underrepresented, as seen in Figure 4.

### 3.4. Comparison of PET/CT and CMR

A total of 65 patients underwent both PET/CT and CMR, for which a Cohen’s Kappa analysis was conducted to assess concordance between the uptake of FDG and LGE on PET and CMR, respectively. A total of 2 segments exhibited substantial concordance, while 13 segments demonstrated moderate concordance. Notably, no segments indicated discordance, as illustrated in Table 6.

### 3.5. Histopathological Evaluation

A total of 16 patients underwent endomyocardial biopsy, with 14 demonstrating evidence of non-caseating granulomas, constituting 15.2% of the overall sample. In addition, 48 patients (52.2%) exhibited positive extracardiac biopsies. Three patients had both positive cardiac and extracardiac biopsies.

### 3.6. Diagnosis and Classification: Definite, Probable, and Possible Cardiac Sarcoidosis

Based on the HRS guidelines, 14 (15.2%) patients were identified as definite cardiac sarcoidosis, 45 (48.9%) as probable cardiac sarcoidosis, and 33 (35.9%) as possible cardiac sarcoidosis.

### 3.7. Systemic Involvement

A total of 4 (4.34%) patients were identified as having isolated CS, 45 (48.9%) had CS with lung involvement, and 14 (15.2%) had CS with skin involvement.

### 3.8. Immunosuppressant Management

A total of 73 (79.2%) patients were treated with immunosuppression. A Fisher’s exact test was conducted to evaluate the association between immunosuppressant therapy initiation and HRS diagnosis categories (Table 7). Patients with a diagnosis of definite cardiac sarcoidosis were 2.447 times more likely to be initiated on immunosuppressive therapy (*p* = 0.019), while those with possible cardiac sarcoidosis had 1.528 times lower odds of starting therapy. In contrast, albeit not statistically significant, a diagnosis of probable cardiac sarcoidosis resulted in reduced odds of commencing therapy. More importantly, there was no association between immunosuppressive therapy initiation and type of sarcoidosis (Table 8).

### 3.9. Major Adverse Cardiac Events

Logistic regression analysis demonstrated that patients with a combination of LGE, FDG uptake on PET, and a “mismatch pattern” exhibited a two-fold increase in the risk of major adverse cardiac events (OR = 2.311, *p* = 0.077).

## 4. Discussion

This single-center study explores the role of advanced cardiovascular imaging in patients with cardiac sarcoidosis. More importantly, we describe its influence on clinical management, particularly on the commencement of immunosuppressive therapy. Prior research has demonstrated the presence of basal septal granulomatous infiltration; our study reaffirms these findings through increased FDG uptake or the presence of LGE in the basal septum [9]. These results are largely consistent with existing literature and further highlight the importance of basal septal inflammation, fibrosis, and wall motion abnormalities as seen on echocardiographic imaging. A significant finding of this study is the concordance between FDG uptake in the mid-anteroseptal and inferoseptal walls and the presence of LGE. Furthermore, patients displaying a combination of the “mismatch pattern” and LGE demonstrated an elevated risk of major adverse cardiovascular events, with an odds ratio of 2.311 (*p* = 0.77).

Although both CMR and FDG-PET are of paramount importance in the diagnosis and clinical assessment of CS, EMBx remains the gold standard for the definitive diagnosis of cardiac sarcoidosis. The clinical utility of EMBx is constrained by several factors, including its limited availability and reduced sensitivity resulting from the heterogeneous distribution of granulomas. Moreover, the procedure is associated with complications, such as pericardial effusion, arrhythmias, and conduction disturbances, which have been documented in as many as 5.5% of patients [8,15]. The transient nature of CS similarly restricts the diagnostic capability of EMBx, akin to that of FDG-PET. In contrast, CMR and FDG-PET are rapid and typically do not necessitate external facilities for interpretation, thereby permitting quicker diagnosis. Furthermore, EMBx offers only limited snapshots of endomyocardial architecture dictated by the specific tissue sampled, while CMR provides a comprehensive assessment of myocardial scar burden, yielding prognostic insights related to the overall area of LGE. As our comprehension of the natural history and localization of cardiac sarcoidosis evolves, it is imperative that we also refine our diagnostic criteria. While EMBx may retain utility in particularly complex cases where results from CMR and FDG-PET are inconclusive regarding suspected isolated cardiac sarcoidosis, it is advisable to prioritize the imaging-based diagnostic criteria established by the HRS and JCS for the accurate and efficient diagnosis of this condition.

FDG-PET and CMR imaging findings have the potential to guide treatment decisions in patients with suspected CS. This is particularly important regarding the timely initiation of immunosuppressive therapy. Given the often rapidly progressive nature of cardiac sarcoidosis, delays in immunosuppression risk irreversible myocardial damage, increase the likelihood of major adverse cardiac events, and ultimately compromise the quality of life for affected patients [2,16]. In cases where imaging findings are highly suggestive of cardiac sarcoidosis, management should proceed proactively to prevent further complications. This strategy is particularly vital for cases where EMBx is not possible or available (cases of probable or possible CS) or in cases of known sarcoidosis where there is a change in clinical state. In these situations, imaging results may support the decision to commence immunosuppressive treatment in the absence of conclusive biopsy results. Notably, this practice was not reliably observed in our cohort, where cases of biopsy-proven cardiac sarcoidosis exhibited a twofold higher likelihood of receiving immunosuppressive therapy and device implantation compared to those identified solely through imaging findings. As multimodal imaging data become increasingly accessible and our comprehension of the pathophysiology of CS improves, the clinical emphasis should transition towards the preservation of cardiac function through the prompt initiation of immunosuppressive therapy in the right clinical context rather than prioritizing a histologically confirmed diagnosis. While this study does not specifically investigate the long-term consequences of late initiation of therapy, future randomized controlled studies should evaluate the prognostic benefit of early imaging and treatment initiation. Wand et al. have shown an association between the mismatch pattern on PET and LGE on MRI with an increased risk of lethal arrhythmias, and that both imaging modalities can be used to prognosticate further outcomes of CS, despite limited guidelines [17].

CMR and PET provide a robust diagnostic framework for identifying CS, specifically focusing on inflammation and scarring. Equally important, by permitting periodic imaging, they facilitate longitudinal monitoring of disease progression and treatment effectiveness over time, in a safe and accessible fashion. Perez et al. highlight this point in their comprehensive review of cardiac sarcoidosis, recommending that all patients with clinical syndromes suggestive of CS should undergo multiple modalities of advanced imaging to help establish a new gold standard for diagnosis, as well as monitor the currently mostly anecdotal treatment strategies. They elaborate further, noting that both CMR and PET are more than twice as sensitive as current diagnostic criteria, and can be more readily managed within the outpatient context [18]. There is growing consensus that TTE, CMR, and FDG-PET, as well as more advanced hybrid imaging, can provide valuable insight into the cardiac involvement in each CS case, allowing for a personalized treatment approach [17,18].

There are several limitations to this study. For instance, being a retrospective and single-center study limits its generalizability, restricting the robustness of subgroup analyses. Moreover, an increased risk of MACE in patients with concerning features suggested by PET and CMR may have reached statistical significance with adequate statistical power. These findings would benefit from confirmation through larger, multicenter prospective studies. Furthermore, despite the implementation of standardized imaging protocols, the possibility of measurement error and interobserver variability in the interpretation of FDG-PET and CMR results cannot be entirely excluded. The reliance on diagnostic coding and imaging findings introduces a risk of misclassification, particularly in cases classified as “possible cardiac sarcoidosis”. Additionally, confounding factors, including coexisting medical conditions and variations in clinical management practices, may have impacted the observed associations. More importantly, given the limited sample size, the study may be underpowered to detect certain differences, raising concerns about Type II errors. Conversely, the multiple statistical tests performed enhance the risk of Type I errors, despite efforts made to mitigate this risk through predefined significance thresholds. These limitations underscore the necessity for future multicenter, prospective studies that incorporate larger sample sizes and standardized protocols to validate these findings. Future research should also focus on longitudinal imaging data to monitor disease progression utilizing advanced imaging modalities and investigate whether there is a temporal aspect to the concordance or discordance of findings from PET and CMR. Furthermore, the prognostic significance of PET and CMR findings in cardiac sarcoidosis patients in whom biopsy is not performed should be compared to those with biopsy to further evaluate the utility of multimodality imaging in CS.

## 5. Conclusions

In conclusion, employing both PET/CT and CMR may provide a safer and more efficient means of diagnosing CS, with future research focusing on monitoring disease progression while evaluating whether it may supersede EMBx as the gold standard for the diagnosis of CS.

## Figures and Tables

**Table 1:** Patient characteristics.

Patient Characteristics	*n* (%)	Mean ± SD
**Demographics**		
Age	92	62.0 ± 11.9
Gender		
*Female*	31 (33.7%)	
*Male*	61 (66.3%)	
Race		
*Caucasian*	42 (45.7%)	
*African American*	35 (38.0%)	
*Hispanic*	7 (7.6%)	
*Other*	8 (8.7%)	
**Clinical Characteristics**		
BMI	72	29.0 ± 5.6
HTN	69 (75.0%)	
HLD	66 (71.7%)	
DM	27 (29.3%)	
Smoking Status	17 (18.5%)	
Family History of Sarcoid	5 (5.4%)	
**Medical History**		
Myocardial Infarction	7 (7.6%)	
Previous Stent Placement	5 (5.4%)	
History of CABG	3 (3.3%)	
Heart Failure	63 (68.5%)	

**Table 2:** PET/CT characteristics.

PET/CT Characteristics	*n* (%) or Mean ± SD
Underwent PET Scan	80 (86.96%)
Immunosuppression Therapy at Time of Scan	36 (39.1%)
Cardiac Uptake	72 (78.3%)
Mismatch Pattern	42 (45.7%)
Ejection Fraction (EF)	47.69 ± 13.94
Fasting Glucose	97.84 ± 27.87
SUVmax	4.08 ± 2.42
SUVmean	1.94 ± 0.84

**Table 3:** Description of FDG uptake according to different myocardial segments.

	Wall Segment	Frequency of FDG Uptake in Segment	Standardized Residual
	Anterior	35 (38.04%)	***2.55***
	Anteroseptal	35 (38.04%)	***2.55***
Basal	Inferoseptal	36 (39.13%)	***2.71***
	Inferior	24 (26.09%)	0.22
	Inferolateral	44 (47.83%)	***4.4***
	Anterolateral	25 (27.17%)	0.43
	Anterior	17 (18.48%)	−1.24
	Anteroseptal	26 (28.26%)	0.64
Mid	Inferoseptal	29 (31.52%)	1.34
	Inferior	25 (27.17%)	0.43
	Inferolateral	27 (29.35%)	0.85
	Anterolateral	23 (25.00%)	0.01
	Septal	10 (10.87%)	***−2.72***
	Anterior	7 (7.61%)	***−3.34***
Apical	Inferior	9 (9.78%)	***−3.01***
	Lateral	11 (11.96%)	***−2.61***
	Apex	7 (7.61%)	−3.34

Bold and italics denote values that are statistically significant.

**Table 4:** CMR characteristics.

CMR Characteristics	*n* (%) or Mean ± SD
Underwent CMR	77 (84%)
LVEF (%)	48.596 ± 14.374
LVESV (mL)	90.721 ± 54.898
LVEDV (mL)	168.955 ± 67.873
SV (mL)	81.729 ± 24.863
CO (L/min)	5.564 ± 1.558
Late Gadolinium Enhancement	51 (66%)
Maximal LGE (%)	52.618 ± 27.076

**Table 5:** Multinomial frequency analysis of LGE by segment.

	Wall Segment	Frequency of LGE in Segment *n* (%)	Standardized Residual
	Anteroseptal	17 (22.1%)	***6.63***
	Inferoseptal	20 (26.0%)	***9.13***
	Inferior	12 (15.6%)	1.68
Basal	Inferolateral	20 (26.0%)	***9.13***
	Anterolateral	7 (9.1%)	−1.64
	Anterior	7 (9.1%)	−1.64
	Anteroseptal	15 (19.5%)	***4.6***
	Inferoseptal	19 (24.7%)	***8.07***
Mid	Inferior	15 (19.5%)	***4.6***
	Inferolateral	14 (18.2%)	***3.55***
	Anterolateral	6 (7.8%)	***−2.69***
	Septal	4 (5.2%)	***−2.69***
	Anterior	2 (2.6%)	***−4.74***
Apical	Inferior	5 (6.5%)	−1.64
	Lateral	2 (2.6%)	***−4.74***
	Apex	5 (6.5%)	−1.64

Bold and italics denote values that are statistically significant.

**Table 6:** Cohen’s Kappa analysis assessing concordance between segmental uptake of FDG on PET and LGE on CMR.

	Wall Segment	Kappa Value	95% CI
Basal	Anterior	0.412	[0.210, 0.614]
	Anteroseptal	0.521	[0.301, 0.741]
	Inferoseptal	0.463	[0.241, 0.685]
	Inferior	0.389	[0.165, 0.613]
	Inferolateral	0.437	[0.211, 0.663]
	Anterolateral	0.282	[0.08, 0.484]
	Anterior	0.329	[0.101, 0.557]
	Anteroseptal	**0.611**	[0.383, 0.839]
Mid	Inferoseptal	**0.623**	[0.436, 0.810]
	Inferior	0.458	[0.223, 0.693]
	Inferolateral	0.402	[0.167, 0.637]
	Anterolateral	0.272	[0.045, 0.499]
	Septal	0.235	[0.034, 0.436]
	Anterior	0.152	[−0.046, 0.35]
Apical	Inferior	0.342	[0.128, 0.556]
	Lateral	0.122	[−0.078, 0.322]
	Apex	0.294	[0.062, 0.526]

Bold denotes values that are statistically significant.

**Table 7:** Fisher’s exact analysis demonstrating the initiation of treatment based on diagnostic criteria.

Fisher’s Exact	*n* (%)	Odds Ratio	Confidence Interval [Upper, Lower]	*p*-Value
Definite CS	14 (15.2%)	**11.55**	[1.165, 200.946]	***0.019***
Probable CS	45 (48.9%)	2.06	[0.701, 6.449]	0.221
Possible CS	33 (35.9%)	0.221	[0.067, 0.656]	***0.004***

Bold and italics denote values that are statistically significant.

**Table 8:** Association of immunosuppression therapy and type of sarcoidosis.

Chi-Squared	*n* (%) (*n* = 93)	Chi-Square (χ^2^)	df	*p*-Value
Isolated CS	4 (4.3%)	2.461	1	0.117
CS + Lung Involvement	45 (48.4%)	0.9	1	0.343
CS + Skin Involvement	14 (15.2%)	1.619	1	0.203

## Data Availability

Data are contained within the article.
